# Plant Flavonoids on Oxidative Stress-Mediated Kidney Inflammation

**DOI:** 10.3390/biology11121717

**Published:** 2022-11-26

**Authors:** Seba Alsawaf, Fatema Alnuaimi, Saba Afzal, Rinku Mariam Thomas, Ayshwarya Lakshmi Chelakkot, Wafaa S. Ramadan, Rawad Hodeify, Rachel Matar, Maxime Merheb, Shoib Sarwar Siddiqui, Cijo George Vazhappilly

**Affiliations:** 1 Department of Biotechnology, School of Arts and Sciences, American University of Ras Al Khaimah, Ras Al Khaimah P.O. Box 10021, United Arab Emirates; 2 Department of Mathematics and Natural Sciences, School of Arts and Sciences, American University of Ras Al Khaimah, Ras Al Khaimah P.O. Box 10021, United Arab Emirates; 3 Independent Researcher, Dubai P.O. Box 72999, United Arab Emirates; 4 Sharjah Institute for Medical Research, University of Sharjah, Sharjah P.O. Box 27272, United Arab Emirates; 5 School of Life and Medical Sciences, University of Hertfordshire, College Lane Campus, Hatfield AL10 9AB, UK

**Keywords:** plant metabolites, bioavailability, inflammatory markers, reactive oxygen species, antioxidant, renal injury

## Abstract

**Simple Summary:**

Increased stress is often observed in patients with kidney diseases, contributing to renal injury progression. Flavonoids are naturally occurring plant compounds with known health benefits, including antiapoptotic, anti-inflammatory, and antioxidant properties. Flavonoids can protect the kidney by improving antioxidant status, ameliorating excess reactive oxygen species levels, and acting as Nrf2-mediators in generating antioxidant responses in the body. Flavonoids also modulate inflammatory markers, exert anti-inflammatory effects, and protect the cells from apoptotic cell death in the kidney. Interestingly, few clinical trials have reported a direct correlation between a flavonoid-rich diet and better kidney disease prognosis. However, flavonoids have a low bioavailability in the body, making it essential to understand better their molecular mechanism of action. We suggest that a flavonoid-rich diet could have promising nephroprotective effects and beneficial outcomes in treating patients with kidney diseases.

**Abstract:**

The kidney is susceptible to reactive oxygen species-mediated cellular injury resulting in glomerulosclerosis, tubulointerstitial fibrosis, tubular cell apoptosis, and senescence, leading to renal failure, and is a significant cause of death worldwide. Oxidative stress-mediated inflammation is a key player in the pathophysiology of various renal injuries and diseases. Recently, flavonoids’ role in alleviating kidney diseases has been reported with an inverse correlation between dietary flavonoids and kidney injuries. Flavonoids are plant polyphenols possessing several health benefits and are distributed in plants from roots to leaves, flowers, and fruits. Dietary flavonoids have potent antioxidant and free-radical scavenging properties and play essential roles in disease prevention. Flavonoids exert a nephroprotective effect by improving antioxidant status, ameliorating excessive reactive oxygen species (ROS) levels, and reducing oxidative stress, by acting as Nrf2 antioxidant response mediators. Moreover, flavonoids play essential roles in reducing chemical toxicity. Several studies have demonstrated the effects of flavonoids in reducing oxidative stress, preventing DNA damage, reducing inflammatory cytokines, and inhibiting apoptosis-mediated cell death, thereby preventing or improving kidney injuries/diseases. This review covers the recent nephroprotective effects of flavonoids against oxidative stress-mediated inflammation in the kidney and their clinical advancements in renal therapy.

## 1. Flavonoids—An Introduction

Plants are an excellent source of secondary metabolites with diverse pharmacological properties that help in disease prevention and treatment. Flavonoids are plant derivatives with a basic phenylchromane skeleton, and their various subclasses include flavones, flavonols, isoflavones, anthocyanin, flavan-3-ols, and flavanones [1,2]. The flavonoids vary in their chemical structure and the degree of unsaturation, oxidation, hydroxylation, and glycosylation of their heterocyclic ring. Flavonoids represent the third-largest group of natural compounds, following terpenoids and alkaloids, comprising approximately 10,000 compounds [3].

Extensive literature has justified the pharmacological benefits of polyphenols owing to their antioxidant and cardiovascular health benefits, which correlate largely with their structure-activity relationship [4,5]. Moreover, recent epidemiological studies have associated a flavonoid-rich diet with a low incidence rate of kidney disease. However, the biological properties of the flavonoids depend greatly on their bioavailability, and their absorption rate might vary depending on the class of flavonoids [6]. A better understanding of the absorption of flavonoids and their mechanism of action is essential for their use as a potent therapeutic agent against kidney injuries.

### Flavonoid’s Bioavailability and Metabolism

A distinct difference exists between the biological properties of flavonoids observed in vitro and their bioactivity in vivo [6]. Hence, it becomes pivotal to understand flavonoid absorption, bioavailability, and metabolism before resolving the question of bioactivity in vivo [7]. Though limited, a fruitful foray into their roles in renal health has gained recent attention and research focus. Moreover, the absorption and bioavailability of dietary flavonoids could vary considerably [8] and are dictated by the food matrix; the kinetics might vary depending on the chemical heterogeneity of saccharides and other functional groups on the flavan nucleus [9]. Dosage, the administration vehicle, antecedent diet, sex differences, individual genetic properties, and the microbial population also influence flavonoid absorption [10].

Flavonoids enter the human body as glycosides [10] and the less prevalent non-glycosylated forms. The evidence suggest that flavonoid glycosides with saccharides linkage have higher biological activity [11]. Even though the nature of the saccharide and its position are crucial for intestinal absorption, the position of the saccharide determines the mechanisms involved in intestinal uptake [12]. Depending on the bioavailability of a flavonoid, its biological properties, including solubility and ability to move across biological membranes, could vary. Moreover, the conjugation in the flavonoid metabolism could influence properties such as size or mass, charge, and hydrophobicity, affecting their excretion rate (via kidney or liver) and plasma retention time [half-life] [13]. The most abundant metabolic transformation of flavonoids occurs via oxidation, reduction, hydrolysis, and conjugation with sulfate, glucuronate, or *O*-methylation involving the liver, intestinal mucosa, kidney, and other tissues [8]. Therefore, an in-depth understanding of flavonoid biotransformation and disposition in vivo is essential.

**Figure 1:**
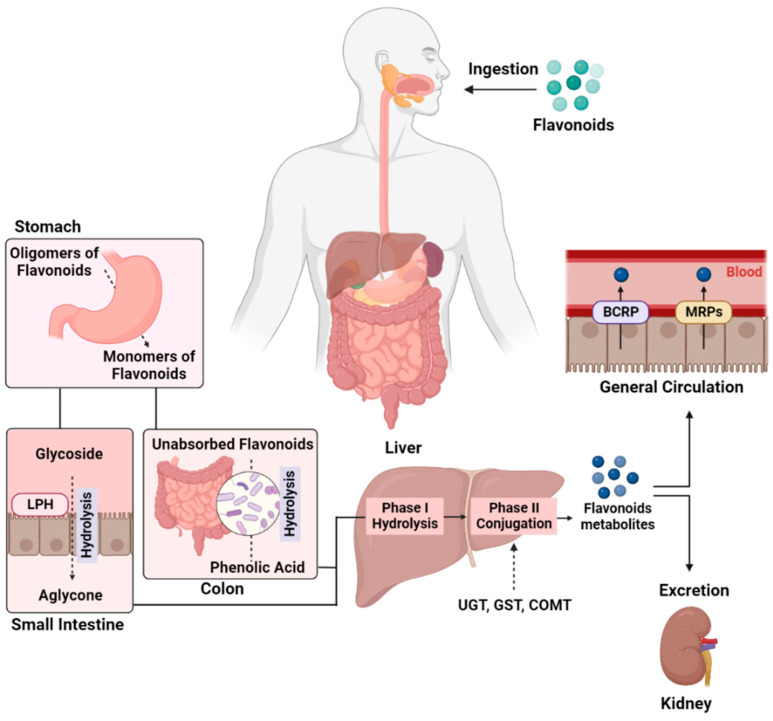
**Bioavailability and metabolism of flavonoids.** After ingestion, flavonoids hydrolyze into monomers in the acidic environment in the stomach. The flavonoid monomers are further metabolized in the small intestine by LPH, which mediates the hydrolysis of flavonoid glycoside into aglycone. The unabsorbed flavonoids transport to the colon and hydrolyze into phenolic acid by gut microflora. These products transfer to the liver through the hepatic portal vein, where phase I (hydrolysis) and phase II (conjugation by enzymes including UGT, GST, COMT) metabolism produce conjugated metabolites. These metabolites deposit into circulation through transporters present on the canalicular membrane of hepatocytes (BCRP and MRPs) to reach the targeted tissues or to be excreted via kidneys in urine.

Lactase-phlorizin hydrolase (LPH) and human intestinal microflora facilitate the hydrolysis of flavonoid glycosides and mark the initial determinant step in the absorption of flavonoids [14] (Figure 1). Flavonoid absorption leads to cytochrome p450-mediated metabolism in the liver through phase I hydroxylation of the aromatic rings. Phase II metabolic enzymes (in the liver), UDP–glucuronosyltransferases (UGTs), sulfotransferase (SULT), and catechol-*O*-methyltransferase (COMT), are essential for flavonoid metabolism, with UGTs being the major contributors, followed by SULTs and COMT; however, the cytochrome P450-mediated phase I metabolism have a minor role [15]. The coupling of transporters (such as breast cancer resistant protein and multi-drug resistant proteins) and phase II enzymes (UGTs and SULTs) plays a significant role in the disposition of flavonoids, especially in the enteroenteric and enterohepatic circulations [14].

For instance, quercetin [16] serves as an excellent example, as its metabolism in humans is well understood, and many of its conjugates have been identified [17,18,19,20,21]. Quercetin exhibits nephroprotective effects both in vivo and in vitro. The molecular and cellular events triggered by quercetin and/or its metabolites in the tubular cells when evaluated by its transit to the kidney, the uptake mechanisms from plasma, the metabolic processes and efflux mechanisms of its metabolites, and their subsequent elimination through urine have revealed its nephroprotective effects [16]. Even though the pharmacokinetics of quercetin have demonstrated its complex migration throughout the body, the metabolic processes that occur in the kidney remain elusive.

## 2. Oxidative Stress—An Overview

Oxidative stress refers to an imbalance in the production and accumulation of reactive oxygen species (ROS) in the cells and the ability of cells to neutralize the reactive products using antioxidant molecules [22]. In low to moderate concentrations, ROS plays a crucial role in cell signaling, gene expression, muscle power regulation, mitogenic responses, apoptosis, and infection resistance [23]. ROS includes superoxide (O2^−^), hydroxyl radical (HO^•^), hydrogen peroxide (H_2_O_2_), peroxynitrite (ONOO^−^), nitrogen oxide (NO^•^), and hypochlorous acid (HOCl). Exposure to exogenous sources such as cigarette smoke, radiation, drugs, smoked meat from cooking, and chemical solvents, when degraded or metabolized, generates free radicals as by-products, leading to excessive ROS production [22,23,24]. An increase in ROS production will disrupt the redox homeostasis, contributing to oxidative stress [23]. Cells protect themselves from ROS-induced cellular damage by recruiting an antioxidant defense system comprising enzymatic components, superoxide dismutase (SOD), catalase (CAT), and glutathione peroxidase (GPx). However, under certain conditions, ROS overproduced cannot be eliminated or neutralized by the antioxidant defense system causing oxidative damage and diseases [23,24]. Superoxide radical (O2^•−^) is a type of ROS generated from the upregulation of nicotinamide adenine dinucleotide phosphate (NADPH) oxidase [24]. Recent studies have shown that NADPH oxidase (Nox)-4 plays an essential role in several kidney disease pathogenesis and is expressed highly in the kidney [25]. SOD catalyzes the conversion of superoxide radicals to hydrogen peroxide (H_2_O_2_), another type of ROS that contributes to renal cell injury [24].

Chemotherapeutic drugs are known to cause an imbalance and induce ROS levels. Doxorubicin (Dox), an anti-cancer drug belonging to the anthracycline family, is often associated with toxic side effects. Dox treatment increases oxidative stress markers while decreasing CAT, GPx, and SOD activities in renal tissue. Further, Dox-induced oxidative stress in renal tissue is characterized by an increased lipid peroxidation marker, malondialdehyde (MDA), and decreased glutathione (GSH) levels. Dox decreases the renal function indicators in urine and serum, including pH, specific gravity, total protein, albumin, urea, creatinine, uric acid, globulin, and blood urea nitrogen (BUN) [26,27]. Frequent use of cisplatin, another chemotherapeutic drug, could also cause nephrotoxicity. Treatment with cisplatin leads to oxidative stress and weakens the functioning of the antioxidant defense system. Increased oxidative stress by cisplatin leads to endothelial damage, impairing endothelial-dependent vascular relaxation [28,29]. A redox-regulated interleukin (IL)-6 protects against cisplatin-induced acute renal failure (ARF) and helps renal recovery by upregulating antioxidative stress factors [30].

### 2.1. Oxidative Stress and Inflammatory Markers in Kidney

#### 2.1.1. Role of Toll-Like Receptor

The Toll-like receptor (TLR) signaling pathway plays a vital role in acute kidney injury (AKI), where the activation of TLRs results in inflammatory cytokines production, leading to renal damage [31]. Inducing AKI by injecting 20mg/kg of cisplatin in TLR2 and TLR4 knock-out (KO) mice showed TLR-2 KO mice with a 20% survival rate and TLR4 KO and wild-type mice with an 80% survival rate with minor differences. Furthermore, the cisplatin-induced renal dysfunction exacerbated the TLR2 KO group with increased serum creatinine, BUN, and urea levels than WT and TLR4 KO mice. TLR2 KO mice also exhibited an exacerbated tissue injury score suggesting structural damage. However, TLR4 KO mice showed no renal injury and were effectively protected, suggesting the role of TLR-2 signaling in cisplatin-induced AKI [32]. It was reported that the absence of TLR4 reduced oxidative stress and increased antioxidant capacity in TLR4 deficient mice compared to WT TLR4 mice. TLR4 deficiency was also linked to decreased inflammation and TGF-β expression [33]. Further, TLR2 increased inflammation, resulting in a lower influx of neutrophils and production of chemokines and TGF- β in the kidneys of TLR2^−/−^ mice compared with TLR2^+/+^ mice [34].

#### 2.1.2. IL-10

IL-10, a cytokine with a general immunosuppressive function, plays a critical role in AKI [35,36]. IL-10 stimulates mesangial cell proliferation and increases the synthesis and secretion of various growth factors, cytokines, and chemokines, causing renal failure [36]. An IL-10 KO (^−/−^) unilateral ureteral obstruction (UUO) model developed to study the effect of IL-10 in renal tubulointerstitial fibrosis demonstrated that IL-10 deficiency deteriorated tubular injuries and promoted renal fibrosis. Moreover, IL-10 deficiency increased the expression of proinflammatory cytokines, tumor necrosis factor (TNF), IL-6, macrophage colony-stimulating factor (M-CSF), and IL-8 as well as chemokines, monocyte chemoattractant protein (MCP)-1 and regulated upon activation, normal T cell expressed and secreted (RANTES) [35]. The impact of IL-10 deficiency on obesity-related renal failure has also been reported previously, where a 12-week high-fat diet caused the IL-10 KO mice to develop severe lipid accumulation in the kidneys, elevated cholesterol, and increased expression of proinflammatory cytokines and chemokines compared to the low-fat diet fed IL-10 KO mice. The mice also presented with high serum creatinine and BUN levels, indicating kidney failure [37]. Similarly, IL-10 KO mice with an ischemia-reperfusion injury (IRI) demonstrated severe renal injury, upregulated AKI markers, and increased proinflammatory cytokines expression. However, treatment with recombinant IL-10 reversed these effects, suggesting the protective effect of IL-10 by suppressing proinflammatory cytokines production and pro-apoptotic factors, and thereby renal dysfunction [38].

#### 2.1.3. IL-6

IL-6 is a proinflammatory cytokine essential for acute phase response to various physiologic insults. A high level of IL-6 was observed in CKD patients and directly correlated to increased oxidative stress [39]. The urine IL-6 levels are demonstrated as an early biomarker for detecting AKI caused by acute tubular necrosis [40]. Consequently, IL-6^−/−^ mice were more resistant to mercuric chloride (HgCl_2_) sensitivity and subsequent AKI when exposed to 5–7 mg/kg HgCl_2_ than IL-6^+/+^ mice. During AKI induction, IL-6 mediates two functions: a cytokine-dependent cell-mediated immune response that aggravates renal injury and a protective response in tubular epithelial cells that alleviate injury, maintaining renal function [40,41]. Active IL-6 signaling has also been demonstrated in cisplatin-induced ARF. IL-6 is produced locally and systemically during kidney injury; while the local IL-6 production protects the kidney from injury during the early phase, the systemic production could result in an AKI [42].

#### 2.1.4. NF-kB

Nuclear factor kappa B (NF-κB) is a transcription factor family that plays critical roles in the initiation and progression of inflammation in pathological conditions. NF-κB can modulate oxidative stress by having anti- and pro-oxidant roles depending on the stress levels [43]. Folic acid (FA)-induced AKI is characterized by increased BUN and serum creatinine, extensive tubular necrosis, loss of brush border, and a significant reduction in mitochondria. Interestingly, FA-induced AKI also demonstrated increased expression of renal p65, NF-κB2, and p53 genes and proteins. Treatment with NF-κB inhibitors and pyrrolidine dithiocarbamate ammonium (PDTC) reversed these effects and improved abnormal renal function. Therefore, simultaneous activation of NF-κB and p53 and the subsequent cross-talk could alter the protective effect of p53 to a proapoptotic role, leading to AKI [44]. Moreover, treatment with dihydroxymethylepoxyquinomicin (DHMEQ) has been effective against renal IRI, with improved serum creatinine levels, reduced levels of the inflammatory cells and inflammatory cytokines, and a decrease in oxidative stress in kidney tissue [45]. Interestingly, recent studies have identified subunits of NFκB to suppress inflammatory responses. The role of the p50 subunit of NFκB, NF-κB1, was studied in an acute and chronic renal injury model, and the results suggested NF-κB1 to have a protective effect during the onset of acute renal injury while not influencing chronic injury [46].

#### 2.1.5. TNF-α

Increased tumor necrosis factor (TNF)-α levels are often associated with renal failure and injury. TNF-α neutralization in rats with renal injury reduced NF-kB activation and inflammatory markers expression and increased NO release, reducing renal inflammation and fibrosis, thereby exerting a protective effect in renal injury progression [47]. Similarly, in the aristolochic acid (AA)-induced nephropathy model of kidney fibrosis, TNF-α inhibition using etanercept (ETN) for eight weeks reduced kidney inflammation and fibrosis, with significant suppression of IL-1β, IL-6, and collagen, suggesting TNF-α as an adjuvant for treating chronic kidney disease with fibrosis [48]. TNF-α is also thought to play essential roles in fatty acid-induced kidney injury by promoting TNF-induced oxidative stress [49].

#### 2.1.6. TGF-β

Transforming growth factor (TGF)-β is essential for normal kidney functioning and contributes to the progression of renal injury. A recent study using SMAD3^−/−^ mice and cisplatin-induced AKI has demonstrated the role of TGF-β/SMAD3 in cisplatin nephropathy. Loss of Smad3 reduced cisplatin-induced kidney injury, inflammation, and Nox4-dependent oxidative stress [50]. An increased TGF-β was also reported in post-contrast AKI (PC-AKI), where an increase in TGF-β resulted in upregulation of TGF-β/SMAD 3 signaling, causing increased fibrosis, decreased proliferation, and increased apoptosis, leading to AKI [51]. Moreover, TGF-β receptor II (TβRII) disruption also prevented impaired TGF−/SMAD-associated severe tubulointerstitial fibrosis in the UUO kidney [52].

#### 2.1.7. IL-1β

**Figure 2:**
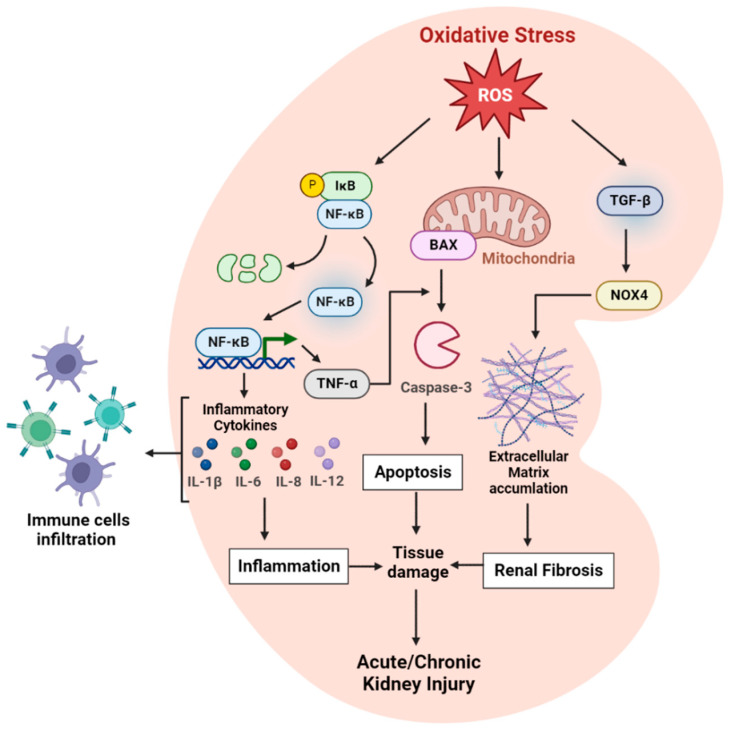
**Oxidative stress and inflammation in the kidney.** Oxidative stress in the kidney is induced by reactive oxygen species (ROS) from mitochondrial, cytoplasmic, and extracellular sources. Under ROS stress, the NF-κB pathway is activated, inducing a proinflammatory response with cytokine secretion (IL-1β, IL-6, IL-8, and IL-12). The cytokines lead to leukocyte infiltration, causing an additional burst of oxidative stress. ROS also increases Bax/Bcl2 ratio in the mitochondrial membrane that activates caspase-3, resulting in apoptosis. In addition, the increased expression of TNF-α by the NF-κB pathway induces apoptosis by forming a death-inducing signaling complex that leads to the cleavage of caspase-3. An increased ROS level activates the TGF-β pathway leading to the extracellular matrix accumulation, causing kidney fibrosis, progressing to renal tissue damage, and acute/chronic kidney injury.

During kidney injury, the immune system releases danger signals. The inflammasome responds to these signals triggering the release of mature IL-1β, which ligates the IL-1R receptor in the kidney cells, implicating the crucial role of inflammasome-IL-1β-IL-1R complex in kidney inflammation and progression to kidney failure [53,54]. An increase in IL-1β is also associated with renal ischemia-reperfusion (I/R) injury resulting in renal dysfunction and is identified as the driving force of renal I/R. Moreover, the expression of IL-1β in kidney tissue increased after renal IR, and the increase in inflammatory stress was associated with increased IL-1β levels [55]. An increased IL-1β was also associated with acute calcium oxalate nephropathy, and inhibiting the expression of IL-1β prevented inflammation-induced renal damage [56]. In cisplatin-induced nephrotoxicity, increased expression of IL-1β was detected, which triggered the activation of IL-1R, inducing TNF-α production and leading to AKI, suggesting that knocking out IL-1 receptor holds the potential of decreasing cisplatin-induced acute kidney injury [57]. The Mechanism of action of oxidative stress-mediated inflammation in acute/chronic kidney injury is shown in Figure 2.

## 3. Nephroprotective Effects of Flavonoids

**Figure 3:**
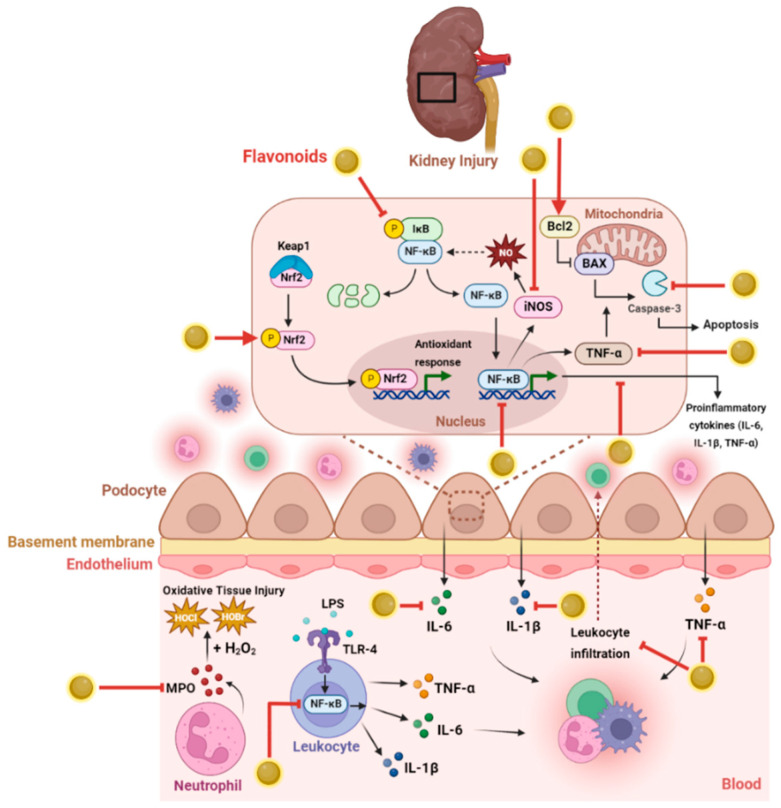
**Mechanism of action of flavonoids in renal protection against oxidative stress, apoptosis, and inflammation.** The first mechanism of flavonoids in response to inflammation involves suppressing the NF-κB pathway by inhibiting the degradation and phosphorylation of the inhibitor of kappa B kinase (IκB), preventing the expression of proinflammatory cytokines (IL-6, IL-1β, TNFα), thereby reducing the infiltration of leukocytes to renal tissues. Flavonoids protect against oxidative stress by decreasing the renal expression of iNOS and the activity of MPO released by neutrophils, which produce reactive species, causing tissue injury. Additionally, the antioxidant effect of flavonoids is exerted by activating the Nrf2 defense pathway, which produces proteins critical in detoxifying and eliminating ROS. In addition, flavonoids have anti-apoptotic properties mediated by increasing Bcl2 levels, which reduces the ratio of Bax/Bcl2, thus preventing apoptosis induction. These anti-inflammatory and antioxidant effects of flavonoids can reverse the process of renal fibrosis and reduce damage to renal tissues.

The protective effects of flavonoids against chemically induced renal toxicity have been widely studied over recent years. Arterial hypertension, oxidative stress, inflammatory diseases, and changes in vascular health could challenge renal health. Flavonoids mitigate these effects and have shown promising results in treating acute and chronic nephropathies, renal fibrosis, and anti-tumor activity [58]. Flavonoids may also act directly on the renal parenchyma and interfere with signaling pathways, affecting the development of renal injury and exerting nephroprotective effects in glomerulonephritis, diabetic nephropathy, and chemically induced kidney insufficiency [58,59]. Flavonoids exert protective effects by decreasing the excessive ROS level or activating renal enzymatic and non-enzymatic antioxidants via different pathways, including modulating the Nrf2-antioxidant pathway [60,61]. Herein, we have assessed and summarized (Table 1) the most reported flavonoids and their nephroprotective mechanisms against oxidative stress, inflammation, DNA damage, and cell death (Figure 3).

### 3.1. Flavonoids in Reducing the Kidney Oxidative Stress

#### 3.1.1. Quercetin

Quercetin, the flavonols found abundant in apples, onion, and garlic, has shown DNA protective effects by alleviating oxidative stress. In ionizing radiation-induced kidney damage, quercetin inhibits neutrophil infiltration and downregulates the subsequent release of proinflammatory mediators, thereby preventing oxidative stress-induced DNA damage and apoptosis [62]. Quercetin also reduces the lead-induced increase in ROS and thiobarbituric acid (TBA) and inhibits oxidative stress, preventing nephrotoxicity [63]. Similarly, quercetin protects against titanium dioxide nanoparticles (NTiO_2_) induced renal toxicity by exerting antioxidant and anti-apoptotic effects [64]. Moreover, in adenine-induced CKD, treatment with quercetin improved renal function by increasing serum LDH, SOD level, and total antioxidant activity, exerting nephroprotective effects [65].

#### 3.1.2. Apigenin

Apigenin is a naturally existing flavonoid found in chamomile, artichokes, celery, and parsley and has demonstrated a protective effect against Dox-induced kidney damage. Dox is a chemotherapeutic agent known to cause substantial damage to the kidney tubule and glomeruli by reducing SOD and GSH activities, and increasing the expression of MDA, thereby increasing ROS production. Treatment with apigenin reversed these effects and inhibited the Dox-induced nephrotoxicity without affecting the cytotoxicity of Dox in tumor cells [66]. Apigenin is also reported to alleviate nanoparticle-induced oxidative stress injury in kidneys. In mesoporous silica nanoparticles-induced nephrotoxicity, apigenin activated the FOXO3a/NF-κβ pathway, resulting in the nuclear translocation of FOXO3a, triggering the transcription of antioxidant genes, subsequently reflecting nephroprotective effects from oxidative stress [67]. Similar protective effects are also reported in nickel oxide nanoparticles-mediated tissue injury, where apigenin increases the renal SOD activity and GSH content, reducing renal MDA levels [68]. These results suggest the capability of apigenin to scavenge ROS through their hydroxyl groups [69]. Exposure to multiwall carbon nanotubes (MWCNTs) induces kidney toxicity by elevating ROS levels, inducing mitochondrial swelling and cytochrome C release. Administration of apigenin decreases oxidative stress parameters and protects against mitochondrial damage [70]. Moreover, apigenin protects against gentamicin-induced renal impairments by restoring enzymatic and non-enzymatic antioxidant proteins, including SOD, glutathione reductase (GR), CAT, GPx, and reduced GSH [71].

#### 3.1.3. Troxerutin

Troxerutin (TRO) is a beneficial, naturally occurring flavonoid in coffee, tea, cereals, and several fruits and vegetables [72]. UUO is known to induce oxidative stress by decreasing SOD levels and increasing MDA levels; TRO restores the normal levels of MDA and increases the activity of SOD, GPx, CAT, and total antioxidant capacity [73]. The protective effect of TRO against drug-induced nephrotoxicity has been studied previously. TRO inhibits the cisplatin and methotrexate-induced oxidative stress by reducing lipid peroxidase and NOX-1, restoring SOD, GSH, and GPx levels, and activating the Nrf2/HO-1 pathway [74,75]. It was reported that TRO could also protect against renal damage caused by nickel cytotoxicity by decreasing lipid peroxidation levels and increasing the antioxidant defense system in the kidney of the rat model [76]. Further, TRO improves renal function, attenuates gentamycin’s effects on renal tissue injury, mitigates gentamycin-induced histopathological changes, and modulates oxidative stress [77].

#### 3.1.4. Epigallocatechin-3-gallate (EGCG)

EGCG is a naturally occurring flavonoid found abundantly in green tea leaves and known for its anti-inflammatory and antioxidant properties. In nanoparticle-induced toxicity, EGCG administration increases the activity of SOD, GSH, CAT, and other antioxidant enzymes, while decreasing the serum MDA and ROS, all of which add to EGCG’s ability to attenuate oxidative stress and damage [78]. Fluoride intoxication increases renal oxidative stress markers and decreases the activation levels of renal enzymatic and non-enzymatic antioxidants; EGCG improves fluoride-induced oxidative renal injury by activating the Nrf2/HO-1 pathway. EGCG treatment decreased the levels of renal oxidative stress markers, enhanced the level of renal non-enzymatic antioxidants like GSH and TSH, and increased the activity of enzymatic antioxidants including SOD, CAT, GPx, GST, and GR [79]. Cadmium exposure increases the ROS and reactive nitrogen species levels, suppress the activities of enzymatic and non-enzymatic antioxidants, including SOD, GR, and GSH, and improve the levels of MDA, GSH, and NO. Treatment with EGCG normalizes the activities of antioxidant enzymes, inhibiting oxidative stress and ultimately reducing the extent of damage caused to the kidneys due to cadmium exposure [80].

#### 3.1.5. Genistein

Genistein is a phytoestrogen obtained from soy-derived foods [81]. Genistein protects against sodium fluoride (NaF)-induced molecular toxicity. Exposure to NaF causes renal damage by elevating intracellular ROS levels, affecting the endogenous antioxidant machinery, and disintegrating the plasma membrane inducing apoptosis. Treatment with genistein protects the cell against defects in cellular homeostasis by inhibiting ROS overproduction and preventing increased MDA levels [82]. The renin-angiotensin system (Ras) is known to induce kidney injury in rats; angiotensin II activates its receptor angiotensin II type 1 (AT_1_R), further activating NADPH oxidase, generating superoxide (Nox4), alleviating renal injury and oxidative stress. Genistein reduced oxidative stress markers and elevated the activities of antioxidant enzymes in hypertensive rats by suppressing the activation of Ras, thereby reducing oxidative stress markers in the renal tissue [83]. In addition, genistein improves antioxidant activity by increasing catalase and total antioxidant capacity and reducing MDA levels and protein carbonyl in kidney tissue of rats with nephrotic syndrome [84]. Exposure to ionizing radiation induces renal injury by increasing MDA levels and decreasing the activity of antioxidant enzymes; however, genistein administration reverses these effects, protecting the kidneys against injuries induced by ionizing radiation and modulating oxidative stress and inflammation. Functional and histologic evaluations for genistein-treated rats demonstrated a significant reduction in ionizing radiation-induced renal injury and reduced renal IRI-induced oxidative stress in the kidney by strengthening the antioxidant system [85].

### 3.2. Flavonoids in Preventing DNA Damage in Kidney

#### 3.2.1. Quercetin

Quercetin was demonstrated to protect against ochratoxin, a mycotoxin that causes severe liver and kidney damage, by increasing the counts of colon *Lactobacilli* and *Bifidobacteria*, crediting to the antigenotoxic and bifidogenic activity of quercetin, thereby reducing ochratoxin-induced DNA damage [86]. Exposing the mice to imidacloprid before treatment with quercetin prevented hepatotoxicity, renal damage, and DNA damage via its antioxidant activity [87]. The effects of quercetin on reducing lead-induced oxidative DNA damage are also reported, where treatment with quercetin markedly lowered 8-hydroxy-2′-deoxyguanosine (8-OHdG), a marker for oxidative DNA lesion following lead exposure in rats, suggesting its potential role in preventing lead-induced oxidative DNA damage in kidneys [88].

#### 3.2.2. Hesperidin

The role of hesperidin in protecting DNA damage induced by sodium arsenite (SA), chlorpyrifos (CPF), and acrylamide (AA) has been reported previously. SA-mediated increase in kidney 8-OHdG was reduced upon treatment with hesperidin, thereby exerting nephroprotective effects [89]. Hesperidin reduces CPF-induced hepatorenal toxicity by reducing 8-OHdG levels and regulating PARP/VEGF genes at biochemical, cellular, and molecular levels [90]. Similarly, AA intoxication can significantly increase 8-OHdG, while administration of hesperidin improves the concentration and activities of antioxidant biomarkers and decreases the expression level of 8-OHdG in kidney tissue [91]. These reports suggest the potential nephroprotective effects of hesperidin against toxin-induced DNA damage.

#### 3.2.3. Naringin

Naringin is a flavone glycoside abundant in citrus fruits like oranges and grapefruit. Naringin is reported to alleviate oxidative DNA damage in acrylamide-induced nephrotoxicity observed by a decrease in 8-OHdG expression [92]. Naringin administration was also reported to protect against CPF-induced nephrotoxicity by reducing oxidative DNA damage in renal tissue. Other nephroprotective effects of naringin include decreasing serum toxicity markers, increasing antioxidant enzyme activity, and regulating apoptosis, inflammation, and autophagy [93]. Similar effects were also observed in 5-FU-induced DNA damage, where naringin exerts an antioxidant effect that reduced the ROS-mediated expression level of 8-OHdG [94].

#### 3.2.4. Chrysin

Chrysin is a naturally occurring flavonoid found in plant extracts and honey and is widely used in traditional medicine. Chrysin reverses DNA damage-induced kidney injuries and exerts a nephroprotective effect. In lead acetate (PbAc) and arsenic-induced kidney damage, chrysin reduces the levels of DNA damage markers and modulators 8-OHdG, COX-2, and KIM-1 compared to the elevated levels detected in untreated subjects [95,96]. Chrysin also alleviates cisplatin-induced renal oxidative damage by eliminating oxidative DNA damage and toxicity markers, including BUN, creatinine, and xanthine oxidase activity, and increasing enzymatic and non-enzymatic antioxidant activity [97]. Further, chrysin pretreatment significantly decreased the number of TUNEL-positive cells with decreased apoptotic and inflammatory markers expression, reflecting chrysin’s ability to attenuate DNA damage and cell death in renal ischemia-reperfusion injuries [98].

### 3.3. Flavonoids Modulate Inflammatory Markers in Kidney

#### 3.3.1. Kaempferol

Kaempferol, a flavonoid found in onions, broccoli, berries, apples, and tea leaves, is known for its anti-inflammatory and antioxidant properties. Kaempferol has demonstrated protective effects against cisplatin-induced kidney injuries. Cisplatin increases the transcription levels of NF-κB, triggering inflammation-related genes and leading to proinflammatory cytokine release, including TNF-α, and IL-12, causing an increase in leukocyte kidney infiltration. However, pretreatment with kaempferol decreases myeloperoxidase (MPO) activity and the release of proinflammatory cytokines, including TNF-α, thereby decreasing the kidney infiltration of leukocytes and alleviating kidney damage. Kaempferol treatment also inhibited the inhibitor of kappa B kinase (IKK) phosphorylation and degraded IκBa, modulating the levels of NF-κB and reducing cisplatin-induced inflammation [99]. Kaempferol also prevents the development of calcium oxalate nephrolithiasis, which occurs as a progression of crystal-induced renal tubular epithelial cell injury. Kaempferol’s role in reducing the deposition of calcium oxalate crystals in renal tubules has been attributed to its suppressive effect on renal androgen receptors (AR) expression. The suppression of AR expression inhibits inflammation and oxidative stress in the kidney by modulating the AR/NOX2 signaling pathway [100]. The involvement of sirtuin-1 (SIRT1) signaling in the nephroprotective role of kaempferol was recently investigated in doxorubicin-treated rats. Pretreatment with kaempferol, followed by doxorubicin administration, protects the kidney’s structure and function by reducing inflammatory markers such as TNF-α and IL-6 [101]. Kaempferol also ameliorates Dox-induced nephropathy by upregulating and activating sirtuin-1 and was associated with increased acetylation of Nrf2 and NF-κB [101]. 

#### 3.3.2. Fisetin

Fisetin is a naturally occurring flavonol with anti-inflammatory properties and is found in several fruits, including strawberries, onions, grapes, and apples. Fisetin exerts nephroprotective effects against cisplatin-induced nephrotoxicity by decreasing the renal protein expression of inducible nitric oxide synthase (iNOS) and reducing the activities of MPO and proinflammatory cytokines. Fisetin was also found to lower the cisplatin-induced DNA binding activity of NF-κB. Moreover, fisetin inhibited the degradation and phosphorylation of IκBα and enhanced the protein expression level of IκBα in kidney tissues. Fisetin administration ameliorated the mRNA expressions of NOX2 and NOX4 induced by cisplatin. These reports suggest the potential role of fisetin in attenuating cisplatin-induced acute renal injury by inhibiting the activation of NF-κB and the subsequent release of proinflammatory mediators [102]. Fisetin alleviates septic acute kidney injury-induced kidney inflammation by reducing the protein expression of IL-6, TNF-a, IL-1β, iNOS, COX-2, and HMGB1. Fisetin inhibits the phosphorylation of transcription factors, including NF-κB, IκBα, and the MAP kinase family members, including ERK1/2, JNK, p38, Src, and AKT [103]. Furthermore, fisetin attenuates hyperuricemia-induced kidney injury by modulating STAT3 and TGF-β signaling pathways. In rats, the elevated TNF-α, IL-6, and MCP-1 were restored within the normal range in fisetin-treated hyperuricemic nephropathy [104].

#### 3.3.3. Luteolin

Luteolin is a natural flavone in several plants, including capsicum, carrots, apple skin, cabbage, onion leaves, and parsley, with potent antioxidant and anti-inflammatory properties [105,106]. PBAc exposure causes an increase in the inflammatory cytokines, IL1β and TNF-α, and the mRNA expression of *Nos2* and *NO* in the kidney tissue. Treatment with luteolin negates these effects by activating the Nrf2/ARE/HO-1 signaling pathway and protects against PbAc-induced kidney injury [107]. The anti-inflammatory and antioxidant effect of luteolin through activation of the NRF2/ARE/HO-1 defense pathway was also observed in bisphenol A-induced nephrotoxicity. Co-administering bisphenol A with luteolin decreased IL-6, IL-1β, and TNF-α levels and was associated with increased Nrf2 accumulation in the nuclear fraction and increased HO-1 levels, suggesting the therapeutic potential of luteolin in bisphenol A-induced kidney toxicity [108]. Luteolin further reduces renal injury caused by lipopolysaccharide (LPS). LPS activates TLR4 signaling pathways, causing upregulation of proinflammatory cytokines and chemokines, including interleukins, IL-1β and IL-6, and MCP-1, and an increase in TNF-α, ICAM-1, and NF-κB. However, pretreatment with luteolin reduces the expression levels of these markers significantly by mitigating the activation of NF-κB and suppressing the release of TNF-α from immune cells and its infiltration into inflammatory cells, causing a significant reduction in interleukin levels and adhesion molecules, reflecting a protective role on the kidneys from LPS-induced AKI [108]. Luteolin also attenuates kidney injury induced by mercuric chloride by exerting its anti-inflammatory effect. Luteolin inhibits NF-κB and Nrf2 signaling pathways leading to a decrease in the expression levels of total TNF-α and the subsequent reduction of other inflammatory markers [109].

#### 3.3.4. Chrysin

Chrysin is identified as effective against paracetamol-induced nephrotoxicity. Paracetamol administration causes a significant increase in TNF-α levels. Treatment with 25 and 50 mg/kg of chrysin decreases TNF-α expression at a percentage level of 15 and 18, respectively. The paracetamol-induced expression of IL-1β and IL-33 was also reduced upon chrysin pretreatment, suggesting that the anti-inflammatory effect of chrysin poses a beneficial therapeutic potential against paracetamol-induced kidney damage [110]. Chrysin ameliorates adenine-induced chronic kidney disease, which causes inflammation by increasing the plasma concentration of IL-1β and IL-6. Co-administration of chrysin with adenine showed a significant decrease in the levels of TNF-α, IL-1β, sclerostin, and endothelin-1, indicating the ability of chrysin in attenuating adenine-induced chronic kidney disease through an anti-inflammatory mechanism [111]. Chrysin also protects against cyclophosphamide-induced nephrotoxicity via inhibiting inflammatory markers. Co-treatment of chrysin (25 or 50 mg/kg) and cyclophosphamide (200 mg/kg) in male Wistar rats reversed the change in the expression of inflammatory markers caused by cyclophosphamide, including NF-κβ, IL-1β, IL-6, TNF-α, and iNOS implicating the nephroprotective effect of chrysin against cyclophosphamide-induced renal toxicity [112].

### 3.4. Flavonoids in Inhibiting Cell Death Mechanisms in Kidney

#### 3.4.1. Quercetin

Administration of 50 and 100 mg/kg of quercetin in mice reduces renal dysfunction and tubular necrosis caused by copper sulfate by reducing oxidative damage, inhibiting caspase-3 and -9 activity, and reducing the mRNA expression of p53 and Bax, suggesting that quercetin alleviates copper sulfate-induced kidney injury by inhibiting mitochondrial apoptotic pathway [113]. Quercetin also reduced apoptosis in carbendazim-induced injury, a fungicide widely used on crops including cotton, grapes, bananas, peanuts, and mushrooms [114]. Increased exposure to carbendazim (50 mg/kg) causes severe hepatorenal dysfunction; treatment with 20 mg/kg of quercetin inhibited caspase-3 expression, suggesting that quercetin can mitigate carbendazim-induced renal injury via an anti-apoptotic mechanism [115]. Furthermore, quercetin can suppress cadmium chloride-induced renal apoptosis and protect the structure of the tubule and glomerulus in a SIRT-1-dependent manner. Quercetin pretreatment significantly increased the tissue level of Bcl2 while decreasing the total protein levels of cleaved caspase-3, Bax, and Bax/Bcl2 ratio and exerted an anti-apoptotic effect in a SIRT-1 dependent mechanism. Quercetin, therefore, exerts anti-inflammatory, anti-apoptotic, and antioxidant properties by deacetylating multiple transcription factors, including p65, eIF2α, Nrf2, and NF-κB [116].

#### 3.4.2. Hesperidin

Hesperidin has anti-inflammatory, antioxidant, and anti-apoptotic properties, and its protective effects against kidney injury induced by several factors have been widely studied. Hesperidin reduces apoptosis in 5-FU-induced kidney damage. Exposure to 5-FU causes a severe increase in the cytoplasmic caspase-3 expression and 8-OHdG; however, pretreatment with hesperidin reduces and controls the increase in the expression of caspase-3 in the renal tubular epithelium, suggesting an anti-apoptotic potential of hesperidin in attenuating 5-FU-induced kidney damage [117]. Hesperidin was also found effective against cadmium-induced kidney injury. Cadmium causes an increase in Bax and caspase-3 levels and a decrease in Bcl2 levels. Pretreatment with hesperidin reduced the Bax and increased Bcl2 levels, reducing the Bax/Bcl2 ratio. Hesperidin also decreased cleaved caspase-3 expression levels, inhibiting or reducing the induction of apoptosis caused by cadmium administration and attenuating cadmium-induced kidney damage [118]. Hesperidin also reduced the nephrotoxic effects of cyclophosphamide by preventing the increase in Bax/Bcl2 ratio and inhibiting the activation of caspase 3, thereby reducing the apoptotic effect of cyclophosphamide exerted upon kidney tissues [119].

#### 3.4.3. Naringenin

Naringenin reduces the methotrexate-induced increase in expression levels of active caspase-3 in the renal tissue and exerts an anti-apoptotic effect by modulating NF-κB, p53, Bcl-2, Bax, and caspase-3 [120]. Similarly, naringenin administration increases Bcl-2 expression in the renal tissue at >24.7%, suggesting an increased inhibition of the mitochondrial pathway of cell death [121]. Naringenin was also shown effective against renal damage induced by vancomycin. Treatment with naringenin (25, 50 mg/kg) following intraperitoneal administration of vancomycin (400 mg/kg) significantly reduced vancomycin-induced increase in expression levels of caspase-3 and caspase-8. Interestingly, 25 and 50 mg naringenin significantly lowered the caspase-9 activity and inhibited the histopathological alterations caused by vancomycin. Therefore, at 25 and 50 mg/kg concentrations, naringenin acts as a potent nephroprotective agent against vancomycin-induced renal injury [122].

**Table 1:** Summarizes various flavonoids and their nephroprotective potentials in renal therapy.

Flavonoid Types	Treatment Design/Dose	Factor/Chemical Studied	Type of Study	Subjects Involved	Key Observations	Reference
Quercetin	20 mg/kg, ip	Ionizing radiation (IR)	In vivo	Sprague–Dawley rats	Significantly reduced the activities of myeloperoxidase and caspase-3, and the levels of 8-OHdG and TNF-α.	[62]
	25–50 mg/kg, intragastrical once daily for 75 days.	Lead	In vivo	Wistar rats	Reduced ROS and thiobarbituric acid reactive substances (TBARS) by modulating MAPK and NF-κB signaling pathways.	[63]
	75 mg/kg, oral gavage for 7 days	(NTiO_2_) nanoparticles	In vivo	Adult female Wistar rats	Normalized the levels of plasma biomarkers and increased the activities of CAT and SOD. Reduced apoptosis and the levels of MDA.	[64]
	5 or 10 mg/kg/day for 21 days	Adenine	In vivo	Male Wistar rats	Reduced serum levels of parathyroid hormone (PTH) and inorganic phosphate, increased serum LDH, urine protein-to-creatinine ratio, urine antioxidants, and IL-8	[65]
	50 mg/kg/day for 15 days	Ochratoxin	In vivo	Swiss albino mice 2–3 months old	Significantly reduced fecal β-glucuronidase activity and the levels of serum ALP, ALT, AST, LDH, and urea. Reduced DNA damage was detected with a 19.6% decrease in tail length. Reduced macrophage spreading by 5.8%.	[86]
	100 mg/kg by oral gavage for 21 days	Imidacloprid	In vivo	Adult male albino rats	Decreased the concentration of BUN and creatinine. Normalized levels of serum total proteins, globulin, albumin, and A/G ratio.	[87]
	10 mg/kg/day by oral gavage for 70 days	Lead	In vivo	Adult male Wistar rats	Lowered the levels of 8-OHdG, ROS, and GSH/GSSG ratio. Restored the activities of Cu/Zn-SOD, CAT, and GPx, and inhibited apoptosis.	[88]
	25, 50 and 100 mg/kg/day for 28 days	Copper sulfate	In vivo	Male C57BL/6 mice	Inhibited the activities of caspases-3 and−9, reduced the mRNA expression levels of p53 and Bax, and activated the expression of Nrf2 and HO-1 mRNAs. Inhibited the expression of NF-κB, IL-1β, IL-6, and TNF-α, and inhibited mitochondrial apoptosis	[113]
	20 mg/kg/day for 14 days	Carbendazim	In vivo	Adult male Wistar rats	Significantly suppressed the increase in IL-1β, TNF-α, and caspase-3 activity. Reduced the levels of ROS, nitrogen species, and lipid peroxidation.	[115]
	50 mg/kg/daily by oral gavage for 56 days	Cadmium chloride	In vivo	Adult male Wistar rats	Preserved the tubule and glomerulus structure, increased creatinine excretion, reduced the urinary levels of albumin, increased the renal activity of Bcl-2, reduced mRNA levels of CHOP, and the protein levels of Bax, caspase-3, and cleaved caspase-3. It also increased the nuclear activity of SIRT1 and reduced the acetylation of eIf2α and XBP1. Reduced the levels of ROS, TNF, and IL-6.	[116]
Apigenin	125,250,500 mg/kg/day for 14 days	Doxorubicin	In vivo & in vitro	Male BALB/c mice. NRK-52E, MPC-5, and 4T1 cells	Significantly increased the activity of SOD and GSH levels. Reduced tissue levels of MDA, TNF-α, IL-6, IL-1β, NLRP3, caspase-1, and generation levels of intracellular ROS. Increased cellular viability and reduced apoptosis in both MPC-5 and NRK-52E cells but not 4T1 cells.	[66]
	Mice: 40 mg/kg for 1 day, ip Cells: 10 μmol/L for 2-and 24-h	MSN	In vivo & In vitro	BALB/c mice and NRK-52E cells	Upregulated the activity of FOXO3a. Increased antioxidant and IkBα levels and reduced ROS accumulation, inhibited the expression of TNF-α and IL-6, and reduced the nuclear translocation of NF-κB. Protected NRK-52E cells from pathological variations and increased cell viability.	[67]
	25 mg/kg/day for 28 days	Nickel oxide nanoparticles	In vivo	Male Wistar rat	Increased renal SOD activity and GSH content and reduced renal MDA levels. Normalized the levels of Ni. Significantly decreased levels of urea, creatinine, and BUN in serum.	[68]
	10 mg/kg for 14 days	Multiwall carbon nanotubes	In vivo	Male Wistar rat	Protected against changes in the activity of mitochondrial SDH, decreased ROS generation, decreased MMP collapse, with a significant reduction in cytochrome c release in kidney mitochondria, and decreased mitochondrial swelling	[70]
	5, 10, 20 mg/kg/day	Gentamicin	In vivo	Male Wistar rat	Increased levels of GR, GPx, SOD, CAT, and GSH. Inhibited changes in serum BUN, creatinine, KIM-1, and NGAL levels. Upregulated Nfe2I2 and Hmox1 mRNA expression. Decreased levels of IL-1β, TNF-α, NFK-β, Bax, and caspase-3.	[71]
Troxerutin	1, 10, and 100 mg/kg	UUO	In vivo	Male Wistar rats	Increased RBF, SOD, CAT, TAC, GPx activity, and Bcl-2 expression. Decreased serum levels of creatinine, Bax, RVR, MDA, cleaved caspase-3, and TNF-α proteins.	[73]
	75 and 150 mg/kg/day, po for 3 days	Cisplatin	In vivo	Male mice	Significantly decrease serum levels of MDA and BUN, and markedly increased the renal levels of SOD and GPx.	[74]
	150 mg/kg/day	Methotrexate	In vivo	Male Wistar rats	Downregulated the expression of HMGB1, RAGE, NF-κB, TNF-α, and COX-2 which lead to the inhibition of HMGB1/RAGE/NF-κB cascade, resulting in a significant reduction in serum levels of BUN, creatinine, and KIM-1. Increased p-AMPK/total AMPK signal and reduced p-mTOR/total mTOR signal. Decreased NOX-1 and lipid peroxidases while restoring levels of SOD, GPx, GSH, and activated Nrf2/HO-1 pathway.	[75]
	100 mg/kg for 20 days	Nickel	In vivo	Wistar Rats	Significantly decreased the levels of lipid peroxidation and increased the levels of enzymatic and non-enzymatic antioxidants.	[76]
	150 mg/kg/day for 15 days	Gentamycin	In vivo	Wistar rats	Significantly increased the rate of glomerular filtration and decreased the levels of serum creatinine, BUN, and urinary albumin to creatinine ratio. Decreased KIM-1 protein expression and decreased levels of protein and lipid oxidative modulations. It also increased the total antioxidant capacity and GSH levels. Decreased the expression of TNF-α, IL-6, and -10.	[77]
EGCG	10 mg/kg/day iv for 35 days	Aluminum oxide nanoparticles	In vivo	Adult male albino rats	Significantly increased GSH concentration, and CAT and SOD activities while decreasing the level of MDA, creatinine, uric acid, and urea.	[78]
	40 mg/kg/day for 28 days	Fluoride	In vivo	Male albino Wistar rats	Reduced levels of lipid peroxidation and protein carbonylation. Normalized the expression of Nrf2/Keap1 and its downstream regulatory proteins. Upregulated anti-apoptotic proteins such as Bcl-2 and downregulated Bax, caspase-9, caspase-3, and cytochrome c. Decreased KIM-1 protein expression, NO, TNF-α, IL-6, and NF-κB expression levels.	[79]
	100, 200 mg/kg/day for 112 days	CdCl2	In vivo	Male Wistar albino rats	Decreased oxidative stress, normalized levels of E-cadherin, and renal enzymatic antioxidant status. Alleviated the over generation of α-SMA, p-Smad3, TGF-β1, and vimentin. Additionally, decreased the production of miR-21 and miR-192, and improved the levels of miR-29a/b/c.	[80]
Genistein	5–60 µM for 2 h.	Sodium Fluoride	In vitro	Normal kidney epithelial (NKE) cells	Prevented LDH leakage, reduced the percentage of apoptotic cells and maintained intracellular levels of ROS, lipid peroxidation, and GSH: GSSG ratio. Increased the activity of CAT, SOD, GPx, GR, and GST. Decreased the expression levels of the activated forms of caspases -3, -8, and -9.	[82]
	40 or 80 mg/kg for 21 days	Renin-angiotensin system (Ras)/Experimental renovascular hypertension	In vivo	Sprague–Dawley rats	Reduced blood pressure, improved renal dysfunction, hypertrophy of the non-clipped kidney (NCK), and atrophy of the clipped kidney (CK). Restored the levels of CAT, SOD, MDA, and the upregulation of AT_1_R, NADPH, Nox4, and Bax, and downregulation of Bcl2 protein in the CK. Inhibited the overexpression of AT_1_R, TGF-β1, smad2/3, and p-smad3 in NCK. Reduced serum ACE activity and plasma Ang II. Alleviated renal hypertrophy in NCK through AT1R/TGF-β1/SMAD-dependent signaling pathways and renal atrophy in CK by modulating AT_1_R/NADPH oxidase/BCL-2/Bax pathways.	[83]
	40 mg/kg/day for 42 days	Adriamycin	In vivo	Adult male Sprague–Dawley rats	Increased CAT and total antioxidant capacity and reduced levels of MDA and protein carbonyl.	[84]
	15 mg/kg in 1mL 1% DMSO via ip. injection	Renal ischemia/reperfusion	In vivo	Adult male Sprague–Dawley rats	Significant reduction in renal injury. Reduced oxidative stress by strengthening the antioxidant system. Decreased levels of MDA, increased activities of SOD, GPx, and CAT, and decreased gene expression levels of TLR4 and TNF-α.	[85]
Hesperidin	100 and 200 mg/kg for 15 days	Sodium Arsenite	In vivo	Male Sprague–Dawley rats	Reduced levels of 8-OHdG, MDA, urea, creatinine, TNF-α, NF-κB, IL-1β, caspase-3, p53, and IL-6. Increased levels of SOD, GPx, GSH, and CAT.	[89]
	50 and 100 mg/kg for 28 days	Chlorpyrifos	In vivo	Male Sprague–Dawley rats	Reduced levels of 8-OHdG and regulated PARP/VEGF genes at biochemical, cellular, and molecular levels. Upregulated Bcl-2 mRNA expression. Alleviated the degenerative and necrotic changes in kidney histology. Reduced PARP-1 activation and the oxidant status by decreasing MDA levels, and increased antioxidant capacity by increasing SOD, CAT, GPx activities, and GSH levels.	[90]
	10 mg/kg/day for 21 days	Acrylamide	In vivo	Male Wistar albino rats	Decreased serum levels of urea and creatinine, OHdG, TNF-α, IL-1β, IL-6, MDA, NO and increased levels of GSH, GSH-Px, CAT, and SOD.	[91]
	25 and 50 mg/kg/day for 7 days, IP	5-FU	In vivo	Male mice	Decreased levels of MDA, and increased CAT, SOD, GR, and GSH activities. Decreased the expression level of caspase-3 and 8-OHdG.	[117]
	200 mg/kg/day for 28 days.	Cadmium	In vivo	Male Wistar rats	Decreased serum levels of creatinine and urea, improved kidney tissue integrity, and maintained normal levels of cellular antioxidants. Significantly lowered MDA levels, Bax/Bcl2 ratio, and levels of cleaved caspase 3, while significantly increasing the levels of SOD and CAT.	[118]
	100 and 200 mg/kg/day, po, for 10 days	Cyclophosphamide	In vivo	Male albino mice	Prevented the increase in Bax/Bcl2 ratio and inhibited the activation of caspase 3, significantly decreasing levels of serum creatinine and cystatin C, renal MDA, and NO, also, increased the ratio of IL-10/TNF-α.	[119]
Kaempferol	100 and 200 mg/kg/day, po for 14 days	Cisplatin	In vivo	Male Balb/C mice	Blocked IκBα degradation and NF-κB nuclear translocation and its binding to the DNA. Reduced the levels of IL-12, TNF-α, and MPO. Blocked MAPK cascade. Upregulated Nrf-2/HO-1 levels.	[99]
	HK-2 cells: 10, 20, 40 μM Mice: 25, 50 mg/kg	Calcium oxalate crystal (CaOx)	In vitro & In vivo	HK-2 cells and Male C57BL/6 mice	Reduced CaOx crystal deposition in renal tubules and adhesion of crystals to HK-2 cells. Suppressed Nox2 by regulating the expression of AR in vitro and in vivo, decreased the levels of MDA, ROS, and H_2_O_2_ in renal tissue, and increased the levels of GSH and SOD. Significantly increased the mRNA levels of IL-4, IL-10, and Arg1 and reduced the mRNA levels of TNF-α, IL-1β, and IL-6.	[100]
	200 mg/kg for 20 days	Doxorubicin	In vivo	Adult male and female rats	Significantly decreased final body weights, levels of urine volume, rate of urinary flow, urinary Cr, and CrCl. Significantly increased mRNA and the total protein levels of MDA, TNF-α, ROS, and IL-6. However, significantly increased the levels of GSH and SOD. Increased nuclear levels of Nrf2 with a parallel decrease in NF-κB p65.	[101]
Naringin	50 and 100 mg/kg/day for 7 days	Cyclophosphamide	In vivo	Male Wistar rats	Decreased serum toxicity markers, regulated inflammation (TNF-α, NF-κB, IL-6, IL-1β), regulated apoptosis and autophagy (caspase-3, LC3B), regulated oxidative DNA damage (8-OHdG), and decreased serum toxicity markers.	[93]
	100 mg/kg/day for 14 days	5-FU	In vivo	Male adult Sprague–Dawley rats	Decreased the weight of kidneys, significantly increased GSH levels, significantly decreased serum levels of BUN, LDH, and creatinine, TNF-α, IL-6, IL-1α.	[94]
Chrysin	25 and 50 mg/kg/day for 7 days	Lead acetate (PbAc)	In vivo	Sprague–Dawley rats	Reduced levels of urea, creatinine, lipid peroxidation, 8-OHdG, NF-κB, IL-33, TNF-α, PGE-2, iNOS, and Cox-2. Increased the levels of SOD, CAT, GSH, GPx, AQP-1, and nephrine. It also decreased the levels of lead, iron, copper, zinc, and sodium, and increased the contents of calcium and potassium in renal tissue.	[95]
	50 mg/kg/day for 30 days	Arsenic	In vivo	Rats	Increased levels of SOD, CAT, GSH, and GST. Reduced levels of urea, creatinine, urobilinogen, KIM-1, neutrophil gelatinase-associated lipocalin (NGAL), NF-κB, IL-1β, TNF-α, IL-6, Cox-2, TBARS and ROS.	[96]
	25 and 50 mg/kg/day for 14 days	Cisplatin	In vivo	Male Wistar rats	Significantly restored membrane integrity and XO activity, significantly elevated CAT, GSH, GPx, GST, and GR activities. Decreased the activity of BUN. Prevented the elevation of creatinine levels. Protective changes in the morphology of tubular epithelial cells, tubules, and glomeruli were also observed.	[97]
	25 or 50 mg/kg/day for 6 days	Paracetamol	In vivo	Male Sprague–Dawley rats	Decreased the levels of serum creatinine, urea, and MDA. Significantly increased the levels of antioxidant enzymes including GPx, SOD, CAT, and GSH while it significantly decreased the levels of inflammatory markers such as TNF-α, IL-1β, and IL-33. A significant decrease in the apoptotic marker Caspase-3 was also observed. The autophagic marker LC3B was significantly decreased.	[110]
	25 or 50 mg/kg/day for 7 days	Cyclophosphamide	In vivo	Male Wistar rats	Decreased the levels of creatinine, urea, MDA, and hepatorenal deterioration. Improved the activities of antioxidant enzymes including CAT, SOD, GSH, and GPx. Reduced alterations in levels of NF-κB, IL-1β, IL-6, TNF-α, iNOS, COX-2, Bcl-2, Bax and LC3B.	[112]
Fisetin	1.25 and 2.5 mg/kg/day, ip for 7 days.	Cisplatin	In vivo	Male Sprague–Dawley rats	Restored the levels of creatinine, BUN, and histopathological alterations. Reduced the degradation and phosphorylation of IκBα, and blocked the nuclear translocation of NF-κB, which decreased the activities of TNF-α, iNOS, and MPO. Impaired the translocation o cytochrome c from the mitochondria to the cytosol which decreased the expression of Bax, cleaved caspases -3 and -9, and p53, and prevented the decrease in the levels of Bcl-2. Significantly lowered the mRNA expression of NOX2/gp91phox, NOX4/RENOX, and the activity of NADPH oxidase enzyme.	[102]
	100 mg/kg/day for 3 days	Lipopolysaccharide	In vivo	Male C57BL/6J mice	Decreased levels of serum BUN and creatinine, decreased the injury markers KIM-1 and NGAL, inhibited renal expression of IL-1β, IL-6, TNF-α, COX-2, iNOS, and HMGB1. Reduced TUNEL (+) apoptotic cells and inhibited Bcl-2, Bax, and activated caspase-3. inhibited Src-mediated NF-κB p65 and MAPK signaling pathways.	[103]
	50 or 100 mg/kg/day	Potassium oxonate and adenine	In vivo	Male C57BL/6J mice	Improved renal function, decreased urinary albumin: creatinine ratio, preserved tissue architecture. Reduced the expression of kidney urate transporters including organic anion transporter 1 (OAT1), organic anion transporter 3 (OAT3), urate transporter 1 (URAT1), and ATP binding cassette subfamily G member 2 (ABCG2). Mitigated the secretion of TNF-α, IL-6, and MCP-1. Restore the expression of alpha-smooth muscle actin (α-SMA), fibronectin, and collagen I. reduced the abnormal activation of STAT3 and TGF-β signaling.	[104]
Luteolin	50 mg/kg, orally for 7 days	PbAc	In vivo	Male Wistar rats	Activated the Nrf2/ARE signaling pathway. Increased the expression of CAT, SOD, GPx, and GR. Decreased the levels of serum creatinine and urea, and decreased the expression of IL-1β, TNF-α, and NO. Upregulated the mRNA expression of Nfe212 and Homx1.	[107]
	100 and 200 mg/kg/day for 28 days	Bisphenol A	In vivo	Adult male Wistar rats	Reduced levels of serum creatinine, uric acid, and BUN, and decreased the generation of IL-1β, IL-6, and TNF-α. Inhibited DNA damage and reduced lipid peroxidation. Augmented the expression of Nrf2 and HO-1 by modulating the Nrf2/ARE/HO-1 pathway.	[123]
	40 mg/kg for 3 days	Lipopolysaccharide	In vivo	Male ICR mice	Decreased levels of BUN and serum creatinine, reduced tubular necrosis, NF-κB, TNF-α, IL-1β, cleaved caspase-3, ICAM-1 expression, and MCP-1.	[108]
	80 mg/kg/day for 14 days.	HgCl_2_	In vivo	Male Wistar rats	Reduced the formation of MDA. Increased the level of GSH. Inhibited the activation of NF-κB. Reduced the accumulation of mercury in the kidneys, increase nuclear translocation of Nrf2 and the resulting protein expression of HO-1 and nicotinamide adenine dinucleotide phosphatase: quinone-acceptor 1 (NQO1).	[109]
Naringenin	20, 40, and 80 mg/kg,	Methotrexate	In vivo	Male rats	Reduced the expression levels of creatinine, urea, NO, MDA, IL-6, TNF-α, and active caspase-3 in the renal tissue. Exerted an anti-apoptotic effect by modulating NF-κB, p53, Bcl-2, Bax, and caspase-3. Significantly increased the expression levels of CAT, SOD, GSH, GPx, GR, and GSH.	[120]
	20 and 40 mg/kg/day, orally for 3 days	CCl_4_	In vivo	Male Wistar rats	Improved kidney tissue architecture. Decreased creatinine, urea, and uric acid levels. Increased the expression of Bcl-2. Significantly changed serum metabolic profiling including an increase in stearic acid, palmitic acid, lauric acid, and myristic acid, and a decrease in the levels of alanine, lactic acid, tryptophan, glucose, and glucosamine.	[121]

## 4. Pharmacokinetics and Nephroprotective Effects of Flavonoids

Even though flavonoids exert nephroprotective effects, the pharmacokinetics of flavonoids should be considered when highlighting their biological effects, as the absorption rate, dose-response, exposure time, and metabolism of flavonoids vary significantly. Herein, we have discussed the dose-effect cause of a few flavonoids and their nephroprotective effects in animals. Different doses of quercetin, from the lowest of 5 mg/kg to the highest of 100 mg/kg, have been studied for its nephroprotective effects. Other doses of quercetin analyzed include 10, 20, 25, 50, and 75 mg/kg [62,63,64,86,87,88,114,115,116]. Interestingly, at all doses, quercetin exerted positive changes to the analyzed markers, including BUN, creatinine, LDH activity, inflammatory, and oxidative stress markers. However, a significant difference was observed to be dose-dependent, with 75 mg/kg and 100 mg/kg showing the most significant alleviation of kidney injury [63,87]. Troxerutin was administered at a range of 1–150 mg/kg. Administration of doses 1 and 10 mg/kg showed no significant improvement, while 75, 100, and 150 mg/kg showed significant inhibition of cell death, inflammation, and oxidative stress in kidney injury models [73,74,75,76,77]. Hesperidin was administered at five different doses (10, 25, 50, 100, and 200 mg/kg) [89,90,91,117,118,119]. All doses affected protein and mRNA expression levels of TNF-α, IL-1β, NF-κB, SOD, MDA, and GSH. However, the groups receiving 25 mg/kg showed no difference against the increase in caspase-3 and 8-OHdG in models treated only with injury-inducing factors, and no significant difference was observed against the before-mentioned markers [119]. The highest efficacy was observed in the group receiving 200 mg/kg [89,118]. The 50 mg/kg and 100 mg/kg groups showed a significant attenuation compared to the group that did not receive any flavonoid treatment. Further, comparing the efficacy of hesperidin at 50 mg/kg to 100 mg/kg showed no significant difference [90]. Genistein was administered at three different dosages (15, 40, and 80 mg/kg). A 15 mg/kg dose of genistein reported a significant reduction in renal injury [85]. The efficacy of genistein against kidney injury was highly proportional to the dosage of administered genistein, i.e., a 15 and 40 mg/kg dose of genistein was sufficient to exert significant anti-inflammatory and antioxidant effects. However, 80 mg/kg of genistein showed more significant efficacy and nephroprotective effects [83].

## 5. Flavonoid’s Clinical Advancements in Renal Therapy

Several clinical trials have been carried out using different flavonoids and natural metabolites as a therapeutic approach to alleviate kidney injuries, including diabetic nephropathy, chronic kidney disease of different stages, end-stage renal disease (ESRD), and glomerular disease. Despite the differences in the causative agent, a common factor shared between the patients is the development of kidney injury that has progressed through similar abnormalities, as detected by changes in the levels of SOD, GPx, NF-κB, TGF-β1, BUN, serum creatinine, catalase, LDH, and proteinuria. Many clinical studies have studied the antioxidant and anti-inflammatory role of flavonoids in treating kidney diseases, a few of which are discussed below.

Baicalein is a flavonoid abundantly found in the leaves and stem bark of the Scutellaria plant, also known as the skullcap herb, and has demonstrated anti-inflammatory, antioxidative, and anti-apoptotic properties [124]. In a randomized controlled study, 95 patients diagnosed with diabetic nephropathy were randomly divided into treatment and control groups and received 800 mg of baicalein three times/day, and a placebo, respectively, for six months. The results showed that baicalein distinctly reduced the activity of aldose reductase (AR), 24 h urinary microalbumin, urinary B2-microglobulin (B2-MG), and urinary albumin excretion rate (UAER). Moreover, a significant increase in SOD and GSH levels and a decrease in NF-κB content and vascular endothelial growth factor (VEGF) were also observed in treatment groups. The administration of the Baicalein flavonoid improved kidney function and delayed the progression of diabetic nephropathy through its antioxidant and anti-inflammatory effects [125].

In another randomized, double-blind study, 42 patients with diabetic nephropathy with an increase in urinary-albumin-creatinine ratio (UACR) >30 mg/g were divided into two groups and received 800 mg of EGCG (four capsules of green tea polyphenols; GTP) and placebo treatment, respectively, for 12 weeks. Both groups also received the maximum recommended dose of ACE inhibitors for 8 weeks before EGCG treatment. EGCG treatment managed to reduce plasma DKK-1 concentrations without affecting blood pressure and glycemic control, implicating that reducing the levels of DKK-1 could downregulate WNT pathway activation, reducing podocyte apoptosis. The treatment significantly reduced TNF-α and UACR levels; however, due to the limited sample size, no significant change in other factors was detected [126]. However, treatment with green tea polyphenols posed no harm or adverse events to the study subject; larger clinical trials are required to explore the effects of EGCG on kidney injuries.

The effects of Brazilian green propolis flavonoids on inflammation in patients diagnosed with end-stage chronic kidney disease and requiring chronic dialysis were studied in a randomized placebo-controlled crossover study, where the individuals were administered 250 mg/day capsules of Brazilian green propolis for 12 months. The mean proteinuria levels decreased by the first two months but significantly decreased by the end of the 6th month of treatment. Improvement in the mean urinary MCP-1 levels and UACR was observed by the end of the 12th month. However, no significant difference in the estimated glomerular filtration rate (eGFR) was observed between the treatment and placebo groups by the end of the 12th month. Data provided by this clinical trial indicated the therapeutic potential of flavonoids present in Brazilian green propolis [127].

In another meta-analysis to understand the effects of *Astragalus membranaceus* in diabetic nephropathy, it was concluded that in comparison to the control group, astragalus injection had a greater therapeutic impact on DN patients, improving systemic condition (serum albumin level), renal protective effect (BUN, SCr, CCr, and urine protein) [128]. A summary of the above-mentioned clinical trials and other similar studies are described in Table 2.

## 6. Conclusions and Future Perspectives

In this review, we have highlighted the recently reported effects of flavonoids and their potential use in treating or improving kidney diseases/injuries. As in the case of many other diseases, flavonoids exert a nephroprotective effect by improving antioxidant status and ameliorating excessive ROS levels. They also act as mediators to activate the Nrf2 antioxidant response, which reduces oxidative stress. Flavonoids also show anti-inflammatory effects in the kidney by modulating several inflammatory markers. Further, flavonoids may also serve as beneficial to reduce chemical toxicity, as detailed in this review. A few clinical trials have shown a promising effect of flavonoids with a direct correlation with a diet rich in plant-based compounds.

A few studies discussed in this review used intraperitoneal administration of flavonoids and thus bypassed the issue of their absorption and bioavailability. However, the current understanding of flavonoid’s absorption rate and bioavailability could be improved by using combinational therapy (with flavonoids or other natural ingredients), engineering microbiota, efficient delivery using nanocrystal or biochemical modifications, etc., which may enhance the pharmacokinetic parameters resulting in efficient treatment. To this extent, more randomized, controlled clinical trials by consuming flavonoids are also recommended to validate flavonoids’ efficacy to be used in renal therapy.

## Figures and Tables

**Table 2:** Clinical trial data showing the effect of flavonoids on kidney diseases.

Type of Flavonoid/Plant	Treatment Design/Dose	Type of Study	Subjects Studied	Key Observations	Reference
Baicalein extract from the *Scutellaria baicalensis* herb	800 mg every 8 h (×3/day) for 6 months	Random controlled study	95 men and women with diabetic nephropathy 46–64 years old 23.4 ≤ BMI ≤ 26.1	A significant decrease in the 24 h urinary albumin, urinary β2-MG, and UAER. A significant increase in SOD and GSH levels and a decrease in the levels of NF-κB, TGFβ1, and VEGF were observed in the treatment group. The increase in BUN and serum creatinine was also significantly reduced at both doses (25 and 50 mg/kg baicalein). It also prevented the increase in the relative weight of the kidney and body’s weight loss.	[125]
EGCG from green tea	800 mg/day for 12 weeks	Randomized, double-blind study	47 patients with diabetic nephropathy Age ≥ 18 UACR > 40 mg/g	Urine Albumin to Creatinine Ratio (UACR) levels were reduced compared to median baseline value. No significant change in blood pressure, BMI, HbA1c, eGFR, or serum CRP was observed. The mean TNFα, and serum DKK-1 levels were reduced.	[126]
Standardized *Aronia melanocarpa* extract (SAE)	400 mg of polyphenols/30 mL/day for 30 days	Clinical trial	30 patients with chronic kidney disease on dialysis treatment	Increase in the levels of hemoglobin, haptoglobin, and LDH levels. Significant decrease in iron, and ferritin, and superoxide anion radical levels, and a decrease in nitrite levels were observed. No significant change in hydrogen peroxide level was observed. The CAT activity was increased, and GSH level was reduced. No significant changes in C-reactive protein, leukocytes, and TNF-α after treatment was observed.	[129]
Aglycone (Daidzein, Genistein) from soy protein	26 mg/non-dialysis day and 54 mg/dialysis session for 8 weeks	Randomized, double-blind study	32 patients with end-stage renal disease on chronic hemodialysis (HD) CRP > 10.0 mg/L	Increase in blood isoflavone levels 5-to10-folds. Serum isoflavone levels correlated positively with the variation of albumin and insulin-like growth factor-1 A trend towards lower levels of CRP was observed.	[130]
*Abelmoschus Manihot (A Manihot)*	2.5 g/×3/day for 24 weeks	Randomized, controlled, clinical trial	417 patients with glomerular disease	The 24-h proteinuria level considerably dropped (*p* < 0.001) upon treatment. No significant difference in eGFR after 24-week treatment was observed. No significant change in serum creatinine levels was observed after treatment. Change in SBP showed a significant difference between the A Manihot and combined treatment groups.	[131]
*Astragalus membranaceus (A membranaceus)*	2.5 g/×2/day for 3 months	Clinical Trial	35 patients with CKD stages 4 & 5	BUN Levels were increased, Levels of eGFR were increased in CKD stage 4 patients, with no significant change in CKD stage 5.	[132]
Brazilian green propolis	500 mg/day for 12 months	Randomized, double-blind, study	32 CKD patients 18-90 years old	Levels of proteinuria, UACR, and Urinary monocyte chemoattractant protein-1 significantly decreased. No change in eGFR was observed	[133]
	250 mg/day, in capsules	Prospective trial, open-label 9-week crossover study	37 patients with end-stage CKD on HD (×3/week) 43.4 ≤ Age ≤ 73.8	Reduced inflammation and serum levels of high-sensitivity c-reactive protein (HsCRP).	[127]

## Data Availability

Not applicable.
